# Influence of 1.5 wt.% Bi on the Microstructure, Hardness, and Shear Strength of Sn-0.7Cu Solder Joints after Isothermal Annealing

**DOI:** 10.3390/ma14185134

**Published:** 2021-09-07

**Authors:** Mohd Izrul Izwan Ramli, Mohd Arif Anuar Mohd Salleh, Andrei Victor Sandu, Siti Farahnabilah Muhd Amli, Rita Mohd Said, Norainiza Saud, Mohd Mustafa Al Bakri Abdullah, Petrica Vizureanu, Adam Rylski, Jitrin Chaiprapa, Marcin Nabialek

**Affiliations:** 1 Center of Excellence Geopolymer & Green Technology (CEGeoGTech), Universiti Malaysia Perlis (UniMAP), Jalan Kangar-Arau 02600, Perlis, Malaysia; izrulizwan@unimap.edu.my (M.I.I.R.); sitifarahnabilah@outlook.com (S.F.M.A.); rita@unimap.edu.my (R.M.S.); norainiza@unimap.edu.my (N.S.); mustafa_albakri@unimap.edu.my (M.M.A.B.A.); peviz@tuiasi.ro (P.V.); 2 Faculty of Chemical Engineering Technology, Universiti Malaysia Perlis (UniMAP), Jalan Kangar-Arau 02600, Perlis, Malaysia; 3 Faculty of Materials Science and Engineering, Gheorghe Asachi Technical University of Iasi, D. Mangeron 41, 700050 Iasi, Romania; 4 Romanian Inventors Forum, St. P. Movila 3, 700089 Iasi, Romania; 5 National Institute for Research and Development in Environmental Protection INCDPM, Splaiul Independentei 294, 060031 Bucuresti, Romania; 6 Institute of Materials Science and Engineering, Faculty of Mechanical Engineering, Lodz University of Technology, Stefanowskiego 1/15, 90-924 Lodz, Poland; adam.rylski@p.lodz.pl; 7 Synchrotron Light Research Institute, Muang District, Nakhon Ratchasima 3000, Thailand; jitrin@slri.or.th; 8 Department of Physics, Czestochowa University of Technology, 42-201 Czestochowa, Poland; nmarcell@wp.pl

**Keywords:** bismuth, intermetallic compound, IMC thickness, mechanical properties, isothermal annealing

## Abstract

This manuscript reports the isothermal annealing effect on the mechanical and microstructure characteristics of Sn-0.7Cu-1.5Bi solder joints. A detailed microstructure observation was carried out, including measuring the activation energy of the intermetallic compound (IMC) layer of the solder joints. Additionally, the synchrotron µX-ray fluorescence (XRF) method was adopted to precisely explore the elemental distribution in the joints. Results indicated that the Cu_6_Sn_5_ and Cu3Sn intermetallic layers thickness at the solder/Cu interface rises with annealing time at a rate of 0.042 µm/h for Sn-0.7Cu and 0.037 µm/h for Sn-0.7Cu-1.5Bi. The IMC growth’s activation energy during annealing is 48.96 kJ mol-1 for Sn-0.7Cu, while adding Bi into Sn-0.7Cu solder increased the activation energy to 55.76 kJ mol^−1^. The µ-XRF shows a lower Cu concentration level in Sn-0.7Cu-1.5Bi, where the Bi element was well dispersed in the β-Sn area as a result of the solid solution mechanism. The shape of the IMC layer also reconstructs from a scallop shape to a planar shape after the annealing process. The Sn-0.7Cu hardness and shear strength increased significantly with 1.5 wt.% Bi addition in reflowed and after isothermal annealing conditions.

## 1. Introduction

Eutectic lead-tin solder has a significant impact on interconnecting an electronic packaging in a variety of electronic systems and assembly. However, lead-tin solder has disadvantages in terms of its harmfulness. This is due to the fact that the lead contained in the solder of discarded electronic components is melted by contaminated groundwater and acid rain. Thus, the European Union’s recommendation on the Waste Electrical and Electronic Equipment (WEEE) Directive and the Directive Restricting the Use of Certain Hazardous Substances quickly triggered the electronic packaging’s use of lead [1,2]. Therefore, the Sn-0.7Cu solder alloy is an outstanding choice rather than the classical Sn-Pb solder alloy in electronic devices [3]. However, in electronic devices, the long-term electronic component usage and the on-off rotations of the power supply could affect the solder joint strength. The IMC in the soldered joint will rise up by solid-state diffusion as a result of the thermal condition under this case. Many scholars have conducted experimental studies utilising isothermal annealing to replicate the actual thermal conditions.

An intermetallic compound denotes a solid phase that forms when two or more molten metallic elements are combined and cooled, where the resultant phase possesses a fixed chemical composition. Primary and interfacial IMC are the two kinds of IMC. The main intermetallic compound is found in the solder joint’s bulk microstructure, whereas the interfacial IMC compound is found at the solder-to-copper substrate interface. The most common intermetallic in Sn-0.7Cu solder alloy is Cu_6_Sn_5_ and Cu_3_Sn. In interfacial IMC, a thin and planar of Cu_3_Sn IMC is formed below the Cu_6_Sn_5_ IMC phase since the Cu_3_Sn IMC acquired greater activation energy than the Cu_6_Sn_5_ IMC phase. Hence, it is recommended that adjustments in microstructure can have a big impact on solder joint strength.

Consequently, the information regarding the microstructure changes in solders and joints is essential. In order to construct dependable lead-free solder joints, it is essential to measure the growth behaviour of the IMC during thermal annealing. Here, the IMC layers grow gradually, leading to decreased ductility and defects-like voids forming at the interface. The presence of defects could cause the failure of the solder interconnection. Recently, Bismuth (Bi) elements were reported that might help boost the intensity of the characteristics in the solder joint [1]. During the soldering and annealing process, the Bi influence on the IMC layer in Sn-Ag-Cu/Cu solder joints was investigated [4,5]. Here, it was discovered that the Bi addition could slow the growth tendency of the IMC layer. It was also shown that Bi’s microstructure of the IMCs and precipitates happened due to supersaturation [6]. As discovered by Teoh et al. [2], the Bi addition on Sn-0.7Cu could increase the solder joint’s mechanical strength. The extension of that study regarding the growth of activation energy of Sn-0.7Cu-1.5Bi in various temperature ranges will be discussed in this manuscript. It is noteworthy to investigate the value of activation energy since it can estimate the expansion of the IMC layer. In our previous study [1], the effect of Bi and Ni addition to the microstructure focusing on the primary intermetallic compound (IMC) growth rate during soldering using the in situ imaging technique were investigated. Basic properties of the solder joint after soldering (without thermal annealing) such as wettability and mechanical properties were also investigated. 

Since the interfacial IMC growth is normally known to influence the solder joint strength, this study investigates the microstructure, hardness, and shear strength of Sn-0.7Cu and Sn-0.7Cu-1.5Bi after isothermal annealing. Focusing more on the growth rate and activation energy of the interfacial IMC after isothermal annealing, the interfacial IMC growth mechanism and its relation to the hardness and shear strength of the solder joint were investigated. As discovered in our previous study [1], the limit of Bi solubility in Sn is around 1.8 wt.% maximum, which is why only the 1.5 wt.% Bi is chosen for this study. This is due to the fact that high Bi addition may lead to the brittle properties of solder joint.

## 2. Materials and Methods

In this work, the mixture of Sn-0.7Cu alloy with 1.5 wt.% of Bismuth was fabricated. Sn-0.7Cu ingot was purchased from Nihon Superior, Osaka, Japan, while Bi was gained from Sigma-Aldrich (M) Sdn. Bhd, Selangor, Malaysia. The Sn-0.7Cu solder ingot and Bi were suspended in a furnace at 350 °C for 1 h. Then, the molten solder was stirred and pour into treated steel moulds and cooled to room temperature. With a diameter of 600 µm, the solder balls were formed with a diameter of 2.0 mm puncher from the alloy foils using 30 µm thickness. These solder balls were reflowed in an oven at 250 °C and eventually went through a sieving process to get a uniform solder size. Then, the solder ball was reflowed on the printed circuit board to form a solder joint. After that, these soldered solder joints were aged at 120, 150, and 180 °C for 24, 120, and 240 h, respectively. 

The cross-sectional microstructure was imaged and analysed at the solder joints interfacial. Here, the influence of Bi addition on Sn-0.7Cu solder alloy at three different temperatures was measured. The IMC layer’s average thickness across the joint was ascertained by analysing the thickness of the IMC layer for each subject from three photographs in three separate places. In addition, the element’s distribution was investigated using a µ-XRF experiment at BL6B beamline at Synchrotron Light Research Institute (SLRI), Nakhon Ratchasima, Thailand. The sample was located at a 90° level between the charge-couple device camera and the X-ray camera. The sample was then scanned at a fast rate in the air atmosphere with a step size of 0.05 mm for 30 s exposure time, which was then examined with the PyMca software (5.0.0, European Synchrotron Radiation Facility (ESRF), France).

The microhardness test was investigated via a Vickers microhardness (FV-700, Future-Tech Corp, Osaka, Japan) followed by the ASTM B933-09 standard test. With a 1 kg indenting load for a 10 s dwell time of the consistency, the results will be calculated using five points for each combination. The shear test was performed to investigate the solder’s joint strength after the annealing process. An Instron machine with a strain rate of 2 mm.min^−1^ was used for shear testing. The specification of shear strength was based on the ASTM D1002. For each annealing condition, five samples were examined, and the average shear strength has been measured. The fractography for the fracture surface was also imaged using an SEM (JEOL, Peabody, MA, USA).

## 3. Results

### 3.1. Primary Intermetallic Compound

**Figure 1:**
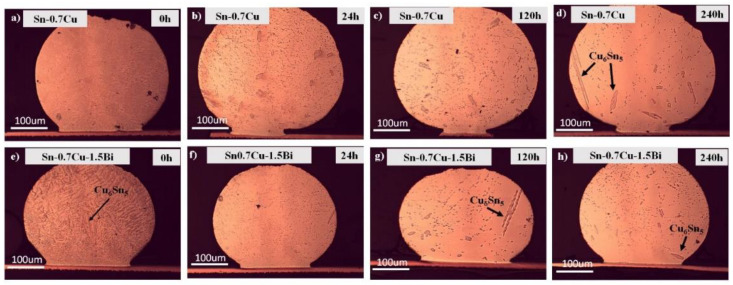
The microstructure of primary intermetallic for (**a**–**d**) Sn-0.7Cu and (**e**–**h**) Sn-0.7Cu-1.5Bi after isothermal annealing at 180 °C.

The microstructure of the primary intermetallic compound of Sn-0.7Cu-1.5Bi as well as Sn-0.7Cu after being aged at 180 °C at various annealing times was portrayed in Figure 1. β-Sn and primary Cu_6_Sn_5_ were found in the Sn-0.7Cu’s bulk solder. In Sn-0.7Cu-1.5Bi solder alloy, the primary IMC did not cause a significant change after the reflowed process. However, after the annealing process at a certain period, it was observed that the hallowed Cu_6_Sn_5_ were formed lesser in comparison to Sn-0.7Cu solder alloy. It is recommended that the dissolution of the Cu atoms in the interfacial reaction solute forms a hallowed Cu_6_Sn_5_ intermetallic during the annealing process [7,8]. In the Sn-0.7Cu-1.5Bi sample, the existing Bi dissolved in the β-Sn phase due to the solid solution mechanism, making the Cu_6_Sn_5_ in Sn-0.7Cu-1.5Bi difficult to be formed.

**Figure 2:**
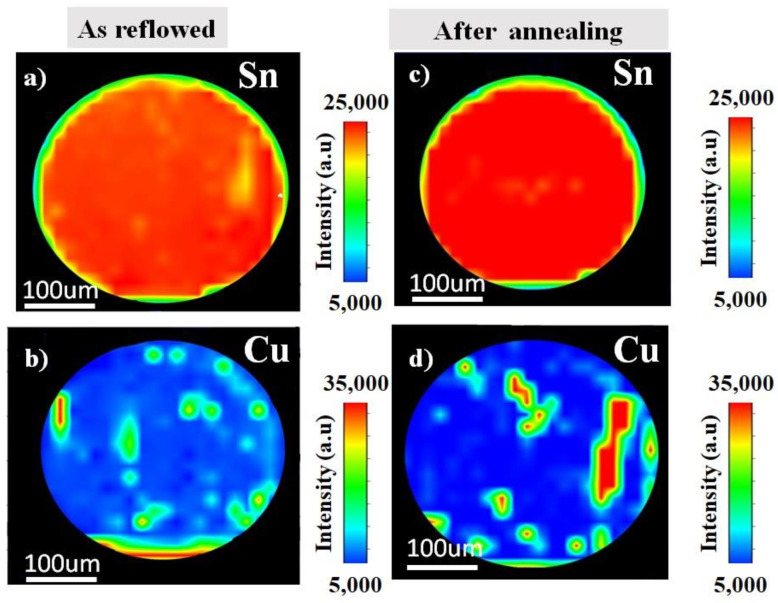
The µ-XRF area mapping of Sn-0.7Cu (**a**,**b**) as reflowed and (**c**,**d**) after isothermal annealing at 180 °C.

**Figure 3:**
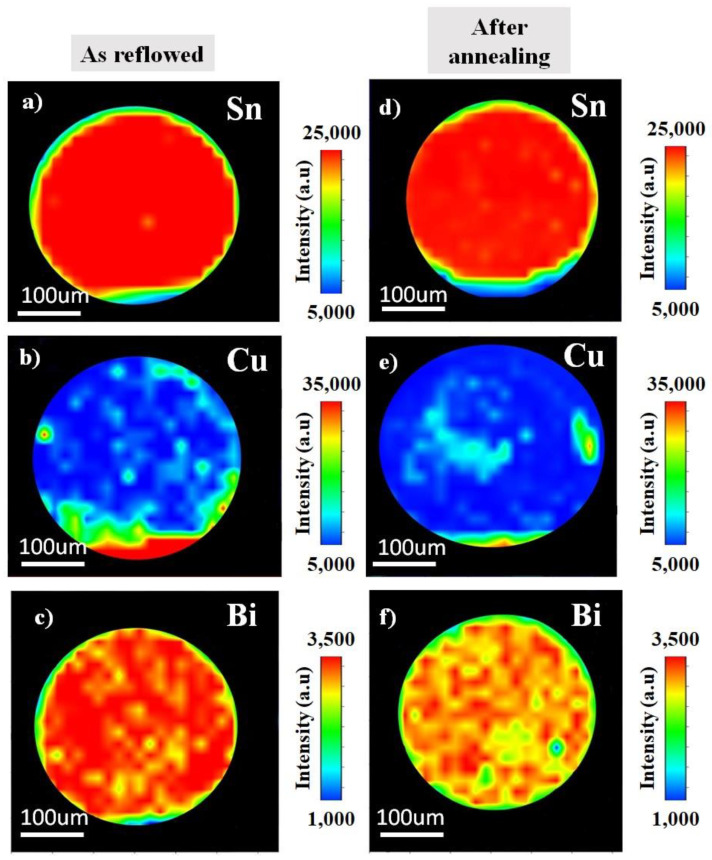
The µ-XRF area mapping of Sn-0.7Cu-1.5Bi (**a**–**c**) as reflowed and (**d**–**f**) after isothermal annealing.

The distribution of the elements as reflowed and after the annealing were analysed using synchrotron µ-XRF mapping. Figure 2 shows the image of an µ-XRF in Sn-0.7Cu solder ball. Here, the elemental map of Sn and Cu as reflowed and after the annealing process are presented. The higher concentration of the element represents the higher intensities. It shows the Sn and Cu dispersion through the Sn-grain. On the contrary, Figure 3 shows the distribution of Sn-0.7Cu-1.5Bi solder balls, indicating the existence of the mapping area of Sn, Cu, and Bi as reflowed and after the annealing process. It shows the lower Cu concentration level in Sn-0.7Cu-1.5Bi compared to Sn-0.7Cu solder alloy, where the Bi element was also found well dispersed in the β-Sn area.

### 3.2. Interfacial Intermetallic Compound and Activation Energy

**Figure 4:**
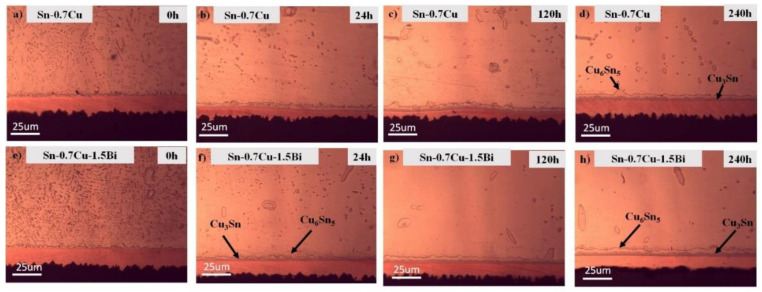
The microstructure of interfacial intermetallic for (**a**–**d**) Sn-0.7Cu and (**e**–**h**) Sn-0.7Cu-1.5Bi after isothermal annealing at 180 °C.

Figure 4 portrays the interfacial microstructures’ evolution of the Sn-0.7Cu/Cu-substrate and Sn-0.7Cu-1.5Bi/Cu-substrate solder joint samples after being aged at 180 °C. It can be seen that the interfacial intermetallic layers constitute Cu_6_Sn_5_ with a round scallop-type morphology and that the Cu_3_Sn layers are too fine to be viewed objectively. In the Sn-0.7Cu system, the interfacial layer commonly consists of two similar layers, which are Cu_3_Sn intermetallic and Cu_6_Sn_5_ intermetallic. The Cu_6_Sn_5_ is normally formed during soldering and grew via diffusion of Cu and Sn reactions, meanwhile Cu_3_Sn grows via diffusion between the Cu-substrate and the Cu_6_Sn_5_ IMC layer. After the reflow process, the interfacial Cu_6_Sn_5_ layers exist in the solder joints in the scallop-like morphology. After the annealing process, this shape gradually changed to planar-like morphology. This total IMC layer thickness also increases significantly, as revealed in Figure 4a–h.

**Figure 5:**
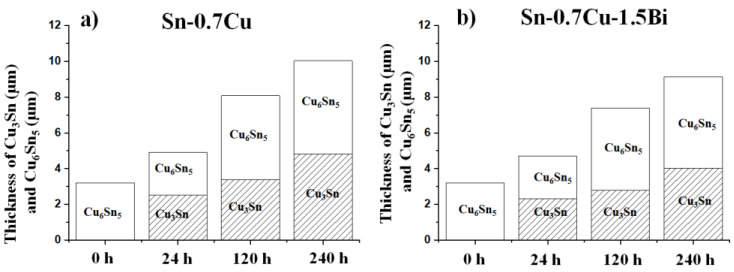
Average thickness of IMC (**a**) Sn-0.7Cu and (**b**) Sn-0.7Cu-1.5Bi during isothermal annealing at 180 °C.

The interfacial intermetallic compound can be divided into two types: The Cu_6_Sn_5_ phase that would grow initially at the soldering interface, then during the Cu_3_Sn phase that grows after the solder joint’s diffusion process [9]. The diffusivity and solubility of Cu in Sn during solid-state annealing are substantially lower than in molten solder. As a result, the IMC growth is substantially gradual, which is amplified when the IMC layer acts as a diffusion barrier [10]. The improvement rate for different phases is distinct, relying on the service requirement. For example, Chen et al. [9] pointed out that the Cu_6_Sn_5_ has a slower rate of growth than Cu_3_Sn under isothermal annealing conditions. Simultaneously, the exaggerated IMC layer’s growth may also deteriorate the solder joints’ reliability [11]. Shen et al. [12] also posited that the IMC layer rises with annealing time due to the solid-state diffusion taking place between Cu atoms from the Cu-pad and Sn from the bulk solder. The average thicknesses of Cu_3_Sn and Cu_6_Sn_5_ at 180 °C are plotted as shown in Figure 5. When the annealing time increases, the Cu_6_Sn_5_ and Cu_3_Sn IMC grows. For Sn-0.7Cu-1.5Bi, the thickness of Cu_6_Sn_5_ and Cu_3_Sn becomes lower relative to the Sn-0.7Cu solder, confirming the fact that the Bi addition effectively retarded the Sn diffusion and prevented the spread of Cu_6_Sn_5_ and Cu_3_Sn. The growth of interfacial IMC thickness (Cu_6_Sn_5_ + Cu_3_Sn) increases with the annealing process at a rate of 0.042 µm/h for Sn-0.7Cu and 0.037 µm/h for Sn-0.7Cu-1.5Bi. The implication also encompasses the fact that the intermetallic compound layer grows gradually and is compacted with the Bi addition.

**Figure 6:**
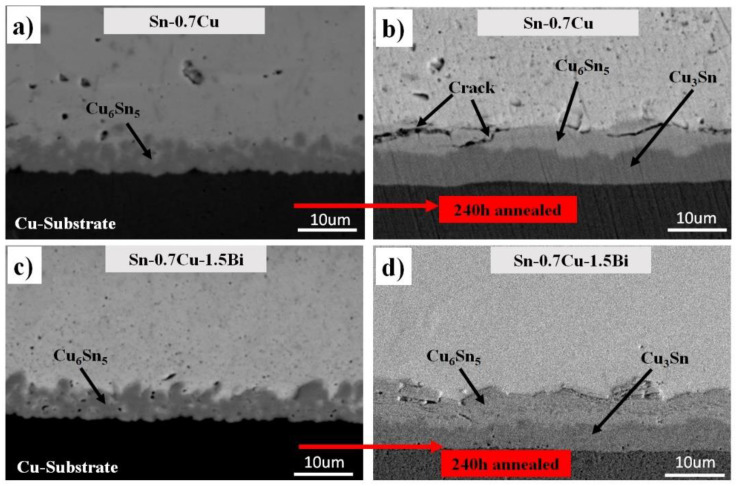
Cross-sectional microstructure of interfacial IMC (**a**,**c**) as reflowed and (**b**,**d**) isothermal annealing at 180 °C.

A cross-section view of the Sn-0.7Cu/Cu-substrate and Sn-0.7Cu-1.5Bi/Cu-substrate solder joints as a reflowed and annealed sample at 180 °C for 240 h is shown in Figure 6. Two types of the interfacial intermetallic compound layer, which is Cu_6_Sn_5_ and Cu_3_Sn was found in the Sn-0.7Cu/Cu-substrate and Sn-0.7Cu-1.5Bi/Cu-substrate solder joints. As compared with the interfacial intermetallic compound of the Sn-0.7Cu-1.5Bi/Cu-substrate solder joints, some cracks were detected in the interfacial intermetallic compound layer of the solder joints at the Sn-0.7Cu/Cu-substrate. This implies that the Bi inclusion could reduce the crack formation at the interfacial intermetallic compound layer. This observation can be clarified with these cracks, which might happen in a thicker intermetallic compound layer formed by higher annealing temperatures due to the inherent brittle behaviour of the intermetallic compound. Impressively, some voids that existed at Cu_3_Sn/Cu and Cu_6_Sn_5_/Cu_3_Sn have an interaction. 

**Figure 7:**
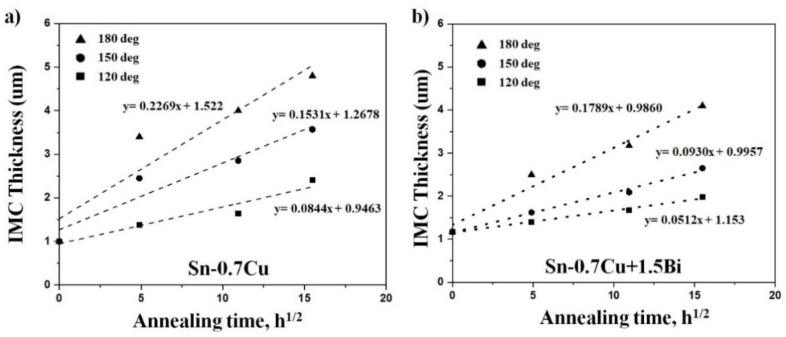
Relationship between the total interfacial intermetallic layer thickness (Cu_6_Sn_5_ and Cu_3_Sn) with annealing time for (**a**) Sn-0.7Cu and (**b**) Sn-0.7Cu-1.5Bi.

In this study, the Cu_6_Sn_5_ and Cu_3_Sn were produced when annealed at 180 °C. The Cu_6_Sn_5_ thickness was increased with the increment of the annealing duration. The 1.5Bi addition, on the other hand, had only a little effect on the development rate of the Cu_6_Sn_5_ intermetallic layer. These values propose that the expansion of the interfacial Cu_6_Sn_5_ layers has been suppressed due to the Bi addition into the solder alloy. It may be surmised that the Bi addition reduced the Sn flux in the Cu_6_Sn_5_ layer due to the diffusion of Sn at that temperature [5]. Therefore, the interfacial IMC thickness for both solders improves as the annealing temperature and duration are increased. The connection between the total interfacial intermetallic compound thickness and annealing period with the Bi inclusion into Sn-0.7Cu is revealed in Figure 7. 

A reflowed intermetallic compound in the solder joint will continue to grow via solid-state diffusion. The growth kinetics after isothermal annealing of the intermetallic layer can be calculated using:

X=√Dt.(1)

Here, X denotes the intermetallic compound thickness at t, while D and t indicate the constant growth rate and annealing time, respectively. The straight-line slope is √D and the growth rate was discovered from a linear regression model of X vs √t.

**Figure 8:**
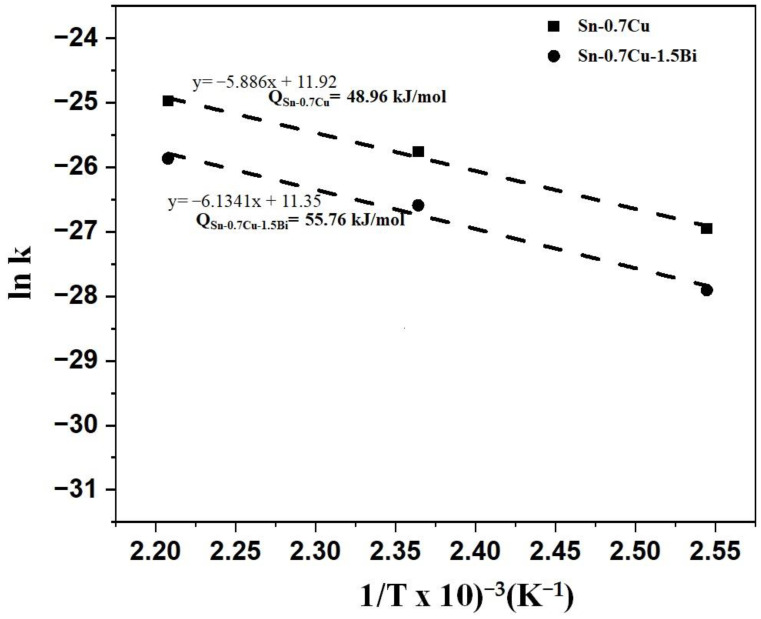
ln k vs. 1/T for the interfacial intermetallic compound growth kinetics for the Sn-0.7Cu-1.5Bi and Sn-0.7Cu solder joints using Arrhenius plots.

The activation energy was measured using the Arrhenius relationship from the layer growth, given by:

(2) $$\;k=k0\exp\;(\frac{-Q}{\mathrm{RT}}),$$

where the diffusion coefficient is k, the constant temperature is k_0_, the activation energy is Q, the gas constant is R, and the absolute temperature value is T. The activation energy could be determined by taking the plot’s slope as shown in Figure 8, as per Equation (3).

(3) $$\;\mathrm{lnk}=\ln k0-(\frac{-Q}{R})\frac{1}{T}\;.$$

The Arrhenius plots of the Sn-0.7Cu and Sn-0.7Cu-1.5Bi of two distinct types of Sn solder joints are portrayed in Figure 8. The activation energy of the intermetallic compound growth during annealing in Sn-0.7Cu is approximated to be 48.96 kJ mol^−1^, while adding Bi into the Sn-0.7Cu solder increased the activation energy to 55.76 kJ mol^−1^. With higher activation energy, it implies that the intermetallic compound needs higher energy to grow. The higher activation energy could be attributed to bulk diffusion via the intermetallic compound layer. The inhibition effect due to the addition of Bi can be interpreted via the following considerations. First, the addition of Bi influences the Sn diffusion’s driving mechanism by the Cu_6_Sn_5_ layer and the formation’s driving force of the Cu_6_Sn_5_ intermetallic [5]. Second, the solid-solution effect with Bi addition causes the lattice distortion of the Sn-rich phase, thus retarding the Sn dispersion from the solder to the intermetallic compound. A mechanism is suggested to elucidate why Bi gathered together to the joint. Regarding the Sn-Bi phase diagram, the Sn-rich phase could dissolve large amounts of Bi at higher temperatures. Through the annealing process, the firstly produced intermetallic compound layers regularly grew to the bulk solder side. The Sn and Cu reactions typically form the Cu_6_Sn_5_ intermetallic compound. The Bi element does not dissipate in the Cu_6_Sn_5_ intermetallic compound. It is initially dispersed in the bulk solder area with an apparent gathering near the intermetallic compound layer. It showed that Bi dissolves typically in the Sn matrix in the Sn-0.7Cu solders. Consequently, Bi gathers in the Sn matrix nearby the joint, precipitating from the Sn-rich phase. It is assumed that the suppression of the IMC layer’s growth rate is due to the precipitation and accumulation of Bi. Generally, the solder alloys with higher activation energies can grow faster at high temperatures and slower at low temperatures. The Cu_6_Sn_5_ phase is typically formed after the reflow process, while the Cu_3_Sn phase is formed via diffusion between Cu and Cu_6_Sn_5_ [13]. This finding focused on the sample that aged at 120, 150, and 180 °C for 24, 120, and 240 h, respectively. However, to better understand the growth behaviour of interfacial IMC, higher temperature and longer time is suggested to investigate.

### 3.3. Mechanical Properties

**Figure 9:**
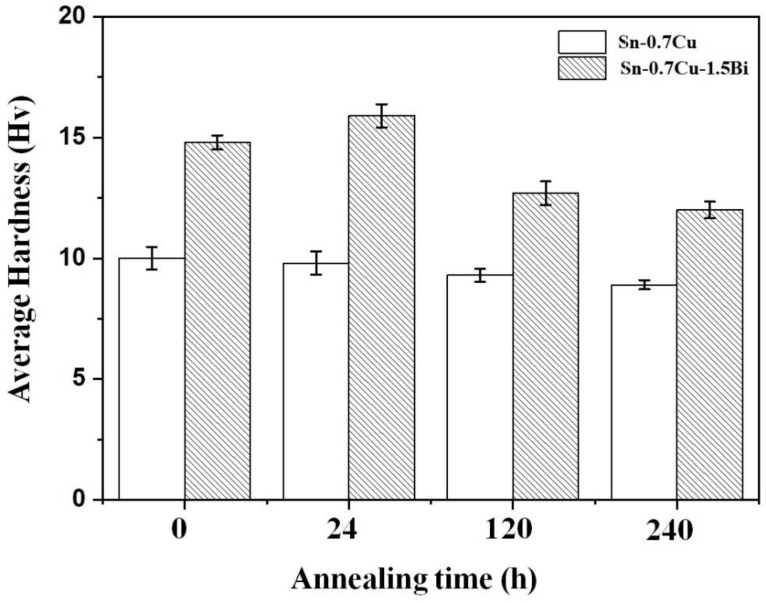
The Sn-0.7Cu and Sn-0.7Cu-1.5Bi hardness after isothermal annealing at 180 °C.

Figure 9 displays the hardness of Sn-0.7Cu and Sn-0.7Cu-1.5Bi solder alloys as reflowed and after the annealing process at 180 °C. The hardness of bulk solder Sn-0.7Cu decreased with the increasing annealing time. Meanwhile, with the inclusion of Bi, the hardness was enhanced by 40.1%. The hardness continues to increase after 24 h annealing. However, it then starts to decrease with 120 and 240 h of annealing time. The hardness was increased due to the Bi addition mainly caused by the coarsening of the microstructure resulting from the IMC’s growth [14]. Bi also strengthened the solder alloy via a solid solution [15]. Due to isothermal annealing, the IMC grew in size. The IMCs are hard but quite brittle, and the formation of a large number of the IMCs in a bulk solder hardened it further. This is caused by the Bi atom addition that can efficiently impede the movement of dislocations, which strengthens the Sn-0.7Cu solder by limiting strain due to the solid-solution effect. The existence of the distributed Bi increased the rate of recrystallisation during the annealing process owing to the increased number of potential sites for nucleation. Contrarily, the distributed Bi acted as the holding agent that retards the grain growth, which resulted in increased hardness. 

**Figure 10:**
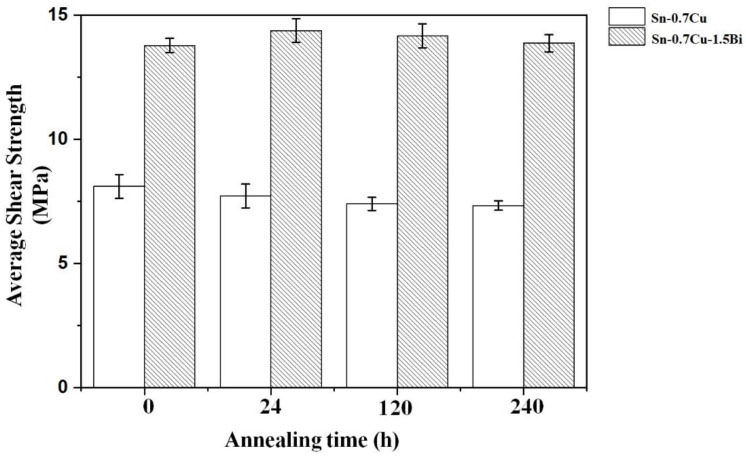
The Sn-0.7Cu and Sn-0.7Cu-1.5Bi shear strength after isothermal annealing at 180 °C.

The shear strength results of Sn-0.7Cu-1.5Bi and Sn-0.7Cu as reflowed and after annealing are shown in Figure 10. The shear test was performed at 180 °C for 24, 120, and 240 h. The results illustrate that the Sn-0.7Cu alloy’s shear strength reduced with an extended annealing time. However, on the Bi addition in Sn-0.7Cu, it showed an improvement of the shear strength of the solder joint by 37%. After annealing samples for 24 h, the Sn-0.7Cu-1.5Bi alloys’ strength increased. However, after annealing for 120 and 240 h, the strength of the Sn-0.7Cu-1.5Bi alloy decreased. This increment in the shear strength of solder alloy was improved with Bi inclusion. This increment via the addition of a metallic element could be ascribed to solid solution hardening. Tateyama et al. [16] pointed out that the addition of 2–3% of Bi renders the solid-solution hardening dominant, which in turn upsurges the shear strength. 

The strength of Sn-0.7Cu after annealing was decreased, which can be attributed to two significant facts. First, annealing coarsens the Cu_6_Sn_5_ intermetallic compounds, which can reduce the capability to prevent the dislocation movement. Second, the β-Sn phase also toughens due to annealing, which decreases the strength. Bi has a solubility limit in Sn at room temperature, and any extra Bi forms a secondary precipitate phase. With the rising temperature, the amount of Bi to enter Sn increased due to the solid solution. Once in the solid solution and at a higher temperature, Bi could diffuse throughout the Sn, which increases the homogeneousness of the alloy. Decreasing the temperature to room temperature forces the precipitation of homogeneously spaced Bi precipitates, contributing to stabilising the strength [17]. Additionally, 1.8% of Bi is considered to have good solubility in Sn at room temperature and can contribute some improvement in strength before the annealing process [18]. 

However, Bi travels into the solution in the β-Sn during the Sn-0.7Cu-1.5Bi alloy’s annealing process. As a result, the solid solution solubility of Bi increases from only 1.8% at 25 °C, to 14% at 100 °C. This suggested that Bi exists in the solidified Sn-0.7Cu alloy’s microstructure as a separate Bi-phase and goes into the β-Sn matrix during annealing, which leads to additional solid-solution strengthening in the Sn-0.7Cu-Bi alloy. We hypothesised that the improvement of Sn-0.7Cu strength through annealing is owing to the improvement in strength from solid-solution strengthening, superseding any decreases in strength due to the intermetallic compound and β-Sn phase coarsening. Ahmed et al. [18] also reported mitigating the annealing effect in SAC-Bi alloys. They reported that Bi atoms are wholly dissolved into the β-Sn induced solid-solution strengthening, thus increasing the shear strength.

### 3.4. Fracture Morphology

**Figure 11:**
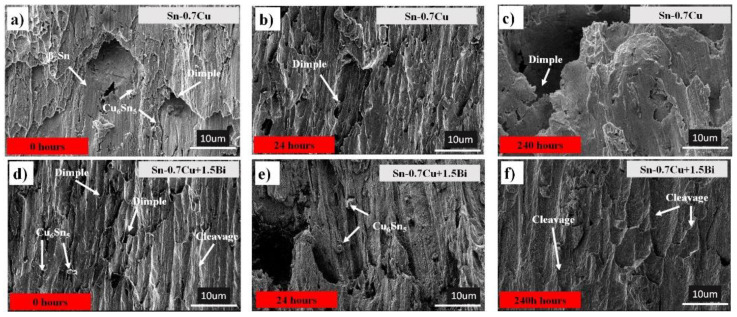
Fractographies of Sn-0.7Cu (**a**) 0 h, (**b**) 24 h, (**c**) 240 h and Sn-0.7Cu-1.5Bi (**d**) 0 h, (**e**) 24 h, (**f**) 240 h.

The fractography of sheared samples was imaged using the SEM to further elucidate the shear behaviours of the Sn-0.7Cu-1.5Bi solder joint. Figure 11 shows the shear fracture morphology of Sn-0.7Cu and Sn-0.7Cu-1.5Bi alloys before and after the annealing process at 180 °C. Figure 11a shows a typical ductile mode on the fracture surface aged at 0 h.

Nevertheless, after Sn-0.7Cu was annealed up to 240 h, the fracture surface changed to a mixture of brittle fracture, as displayed in Figure 11b. Bi (1.5 wt.%) was added to the mix, which showed a ductile fracture mode with a small dimple. After the sample was annealed up to 240 h, it showed a cleaved pattern and brittle failure mode. It also can be seen that the joint break happens at the interfacial IMC when a thicker IMC forms due to the annealing process. Another reason is the existence of an initial crack at the interfacial IMC, as shown in Figure 6b,d. This can be related to the reduced activation energy of IMC in the Sn-0.7Cu solder joint, which increased the IMC’s growth thickness during the annealing process. However, with the Bi inclusion in Sn-0.7Cu, the IMC needs higher energy to grow, which could reduce the IMC thickness growth.

Thus, it was concluded that if the fracture happens at the bulk region, it is ductile [19]. Moreover, if the fracture occurs in the region near the solder and intermetallic interface, it is a mixture of brittle and ductile fractures [20]. As a result, the strength near the solder and intermetallic interface is a weak point for the fracture, resulting in decreased shear strength with time.

## 4. Conclusions

This study presents the mechanical characteristics of Sn-0.7Cu-1.5Bi, which are examined on the primary and interfacial intermetallic after isothermal annealing. Some conclusions were drawn from the results of this work:

1. The 1.5 wt.% Bi addition did not lead to a significant change in the primary Cu_6_Sn_5_, as was observed in the bulk solder Sn-0.7Cu. The µ-XRF analysis shows the lower Cu concentration level of Cu_6_Sn_5_ primary intermetallic with the addition of Bi after the annealing process. It can also be seen that the Bi element is well dispersed in the β-Sn region.
2. The growth of interfacial IMC thickness (Cu_6_Sn_5_ + Cu_3_Sn) increases with the annealing process at a rate of 0.042 µm/h for Sn-0.7Cu and 0.037 µm/h for Sn-0.7Cu-1.5Bi. The shape shifted from the scallop shape to the planar shape after the annealing process with a small microcrack form. Bi also inhibited the growth of Cu_3_Sn interfacial intermetallic compound after the annealing process.
3. The activation energy for the formation of intermetallic layers of Sn-0.7Cu was set to 48.96 kJ mol^−1^, and the activation energy of the Sn-0.7Cu-1.5Bi system was measured at 55.76 kJ mol^−1^. It is obvious that the intermetallic compound thickness of the Sn-0.7Cu-1.5Bi system is difficult to grow when matched to the Sn-0.7Cu system due to the high activation energy.
4. Compared to Sn-0.7Cu, adding Bi improves hardness by 40.3% and shear strength by 37.1%. After 24 h of annealing, the shear strength of Sn-0.7Cu-1.5Bi was increased. This is due to the fact that with the addition of Bi, it can be that the dissolution of the Bi element in the matrix improved the shear performance of the solder joint. However, a longer annealing time could reduce the shear strength. The fracture of the annealing sample shows a mixture of brittle fractures in the area near the solder/intermetallic interface due to a thicker intermetallic compound layer during the annealing process.

## Data Availability

The data presented in this study are available on request from the corresponding author.
